# Clear cell carcinoma arising in previous episiotomy scar: a case report and review of the literature

**DOI:** 10.1186/s13048-016-0211-5

**Published:** 2016-01-12

**Authors:** Ling Han, Ai Zheng, He Wang

**Affiliations:** Department of Obstetrics and Gynecology, West China Second University Hospital, Chengdu, China

**Keywords:** Endometriosis, Clear cell carcinoma, Episiotomy scar, Malignant transformation

## Abstract

**Background:**

Malignant transformation of endometriosis associated with episiotomy scar is a rare event, especially histological type of clear cell adenocarcinoma. There are only three clear cell carcinoma in episiotomy scar reported, no standard treatment established.

**Case presentation:**

A 36-year-old woman presented with a two-month history of painless but puritic perineal lump which she noticed was gradually enlarging. She had undergone surgical excision of a mass in the episiotomy scar 9 year ago and resequently histological type of endometriosis. Physical examination revealed a 10 × 5 cm soft, purple scar which is closely related to the apex of the episiotomy.We underwent a local excision of the mass for a biopsy . The second surgery performed after one cycle of paclitaxel and cisplatin (TP) to permit clearance of tumor while preserving normal vaginal function.Pathological result was clear cell adenocarcinoma. Two cycles of TP adjuvant chemotherapy were administrated after surgery.

**Conclusions:**

We report a case of primary clear cell carcinoma developing within a previous episiotomy scar in a patient with a history of endometriosis, along with a review of the literature. Accumulation of management data on these rare tumors and Long-term follow-up of such patients is therefore important.

## Background

Malignant transformation of endometriosis associated with episiotomy scar is a rare event, especially histological type of clear cell adenocarcinoma. In 1990 Hitti first described primary clear cell adenocarcinoma in a perineal endometriosis. To the best of our knowledge, there are only three case reports on record. We report a case of primary clear cell Carcinoma developing within a previous episiotomy scar in a patient with a history of endometriosis, along with a review of the literature.

## Case presentation

A 36-year-old woman presented with a two-month history of painless but puritic perineal lump which she noticed was gradually enlarging. The patient’s past gynecological history included frequent vaginitis owing to bad health habits and the lack of professional treatment. Because of discomfort, the patient often scratched the vulva including the lesion of the episiotomy scar for many years. In addition, her past obstetric history was significant. She had a history of a forceps delivery 20 years ago. The postoperative recovery of perineal wound was slow. Several months after delivery, she experienced cyclic perineal pain and swelling of the episiotomy scar. Mifesterone and Medroxyprogesterone acetate injectable suspension (DMPA) were used and the pain was relieved. She had undergone surgical excision of a mass in the episiotomy scar 9 year ago and resequently histological type of endometriosis. DMPA was administrated for one year and then mifesterone for half a year. Medical treatment with Chinese traditional medicine was prescribed after that.

**Fig. 1:**
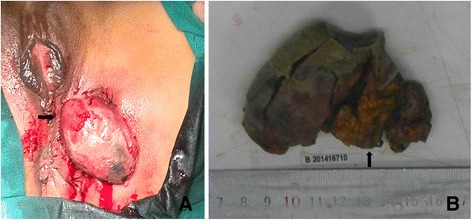
**a** Perioperative photograph showing a 10 × 5 cm perineal mass arising from the episiotomy scar. **b** Cut surface of the tumor, pink-yellow in color

**Fig 2:**
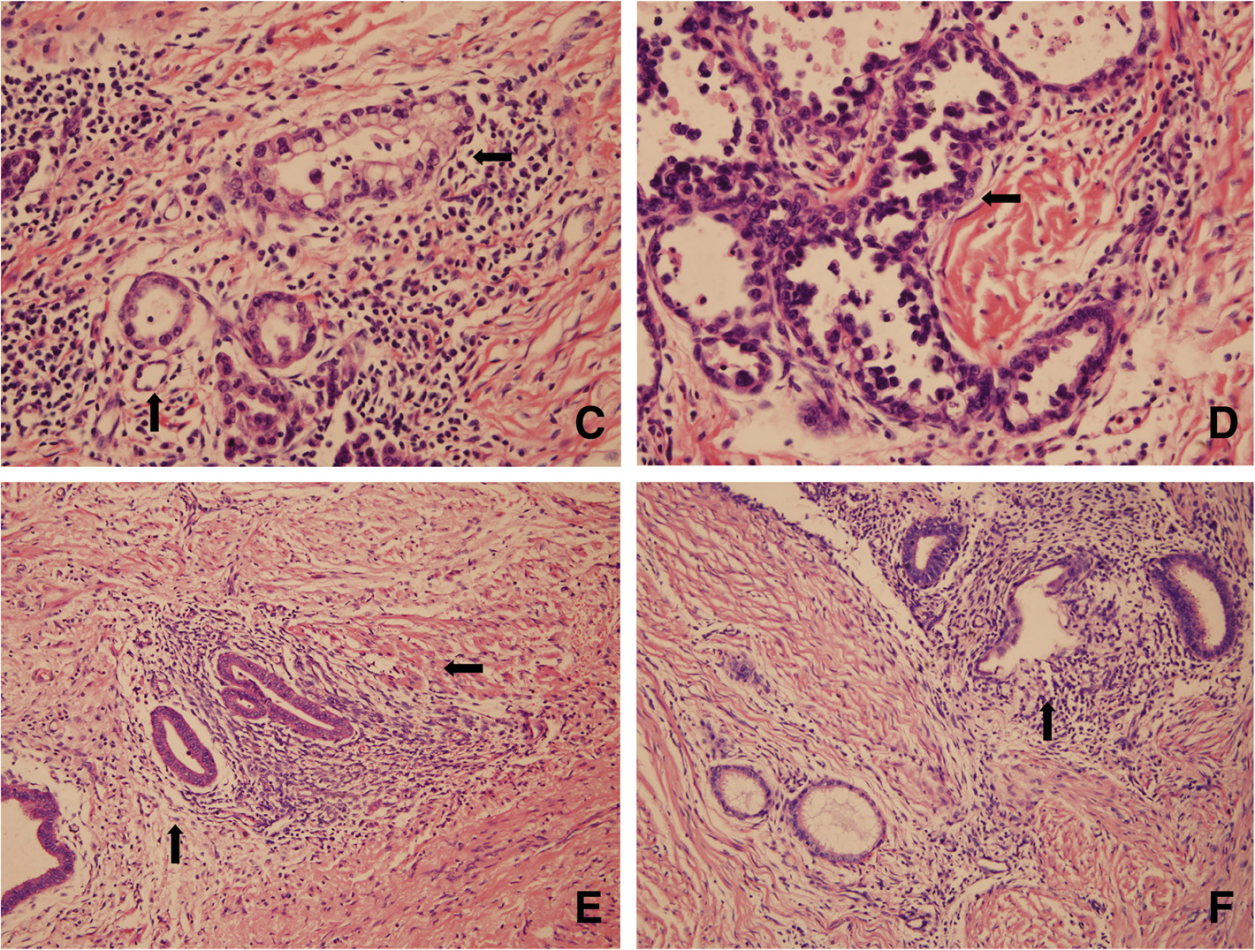
Microscopic images: **c** clear cell adenocarcinoma: marked cellular pleomorphism, clear cytoplasm, nuclei show pleomorphism. **d** Characteristic hobnail cells are noted in our case. **e** Endometriosis: cystic dilated endometrial glands in dense stroma. **f** Transitional zone revealing clear cell carcinoma arising from the endometriotic focus

Physical examination revealed a 10 × 5 cm soft, purple scar which is closely related to the apex of the episiotomy. Pelvic examination and trans-vaginal ultrasound did not detect other signs of pelvic or extra-pelvic endometriosis. We underwent a local excision of the mass for a biopsy (Fig. 1a and b). Microscopically, pathological result was clear cell adenocarcinoma (Fig. 2c and d). Endometriotic focus was seen at the side of the cacinous area (Fig. 2e). We clearly noticed transitional dysplastic zone between the endometriotic focus and the clear cell carcinoma (Fig. 2f). The second surgery performed after one cycle of paclitaxel and cisplatin (TP) to permit clearance of tumor while preserving normal vaginal function. The patient underwent radical vulvar excision with skin graft and inguinal lymphadenectomy. The pathologic result showed the deep and lateral margins are clear. The patient was discharged after 10 days of hospitalization without any complication. Two cycles of TP adjuvant chemotherapy were administrated after surgery. The patient has returned to work at the time of manuscript preparation.

## Discussion

Sampson first proposed three criteria to diagnosis malignancy arising in endometriosis in 1925 [1]. The clinical symptoms of malignancy of endometriosis were menstrual irregularity, the enlargement of the mass, increased pain, etc. There are no specific tumor markers in malignant transformation of extraovarian lesions. J. Cuisenier reported the level of CA125 is within normal range in 44 % of patients with extraovarian lesions versus just 15.38 % of patients in ovarian endometriosis [2]. Patients with endometriosis were reported to be 3-folds associated with clear cell carcinoma and endometroid carcinoma. Because of the high risk and no typical clinical tumor marker, patients those were verified as endometriosis in episiotomy scar should be followed up closely.

Endometrioid and clear cell carcinomas are a more common histotype in endometriosis associated ovarian carcinoma (EAOC) than in non-EAOC. The histotype of malignancy occurring from extraovarian endometriosis was reported as follows: endometrioid carcinoma between 75.9 and 69.1 %, clear cell carcinoma between 13.5 and 4.5 % [3]. Sara C reported 28 cases of malignancy arising from abdominal wall scars, of which 22 were endometroid or clear cell carcinoma [4]. Among four reported malignancies arising from episiotomy scars, three were clear cell carcinoma [3, 5–7]. The presence of a transitional dysplatic zone between benign endometriosis and cancer is one of the most important feature in the microscopic appearance of a carcinoma arising from endometriotic foci [8]. Approximately 36–42 % of endometriosis associated cancers have this dysplatic zone [9]. The previous three cases of clear cell carcinoma arising from episiotomy scar also reported the transitional zone. The transitional zone was detected in our report. In addition, similar to the present case, the microscopic or macroscopic coexistence with endometriosis was reported in the past three cases.

The best explanation of the pathogenesis of endometriosis in episiotomy scar is transport theory. Transport theory involves iatrogenic transplantation of the endometrium to the surgical wound. Our patient had a lateral episiotomy because of dystocia. This theory might explain the endometrial tissue can be transported to the vulvar tissue during delivery. However, this theory can not explain the rare cases of endometriosis in organs such as the lung and kidney [10]. There are other theories such as lymphatic or haematogenic dissemination, coelomic metaplasia and cell immunity change theory, which are proposed to explain this.

Whether or not endometriosis in episiotomy scar is a sign of its progression to carcinoma have a controversy. The rare case of this clear cell carcinoma in previous episiotomy scar may give evidence to tumor carcinogenesis in extraovarian endometriosis. The etiology of malignant transformation of endometriosis is not clear. Several aspects were summarized as the cause of malignant transformation of endometriosis. First, the genetic mutation was proposed to be associated with the transformation of endometriosis into carcinoma. Loss of heterozygosity at locus 10q23.3, mutation of the tumor suppressor gene PTEN and P53 alteration was reported to be relevated with the development of endometriosis associated cancer [11]. Mutations of ARIDIA was reported more often in clear cell carcinoma [12]. David G. Huntsman reported the Mutations of ARIDIA and the loss of BAF250a expression, is an early event in the transformation of endometriosis into cancer [13]. *ARID1A* inactivation and PI3K/AKT pathway alterations may be consistent to initiate carcinogenesis [14]. The detection of gene mutation is difficult in clinical work, but these research give us some potential therapeutic approach in the future.

Secondly, hormone level has a role in the pathogenesis. Hyperestrogenism was reported to be associated with the development of endometrioid cancer and clear cell carcinoma [15]. Okamura K reported progesterone resistance within endometriosis [11]. This can explain why the use of DMPA for a long time in our case and the endometriosis also developed into cancer. In addition to that, Endometriosis is associated with a local inflammatory reaction leading to cytokine release. Balkwill and Mantovany offer an description of this link: If genetic damage is the ‘match that lights a fire’ of cancer, some types of inflammation may provide ‘the fuel that feeds the flames [16]. Cytokines within the endometriosis microenvironment, such as IL-1 is associated with increasing the synthesis of prostaglandin E2 (PGE2) which cause angiogenesis, proliferation, and inhibition of apoptosis similar to malignant mechanisms [17].

Table 1 summarize the previous reported cases arising in episiotomy scar together with our case. A total of 4 cases of malignant transformation of episiotomy scar endometriosis have been reported in previous literature. Table one list several reports with different treatments of these disease. The radical excision was administrated along with or no adjuvant therapy. Because the tumor size of our patient is big and the histological type was clear cell carcinoma, chemotherapy was administrated in our patient and the recovery of the patient was well till now, but long-term follow-up result of the efficacy of adjuvant therapy is uncertain.

**Table 1:** Summary of the carcinoma arising in episiotomy scar

Author	Year	Histology	Treatment	The follow up
Hitti IF [5]	1990	Clear cell carcinoma	radiotherapy and chemotherapy	dead at 30 months
Todd RW [6]	2000	Clear cell carcinoma	radiotherapy and chemotherapy	remission at 6 months
Chene G [3]	2007	Serous papillary Cystadenocarcinoma	Complementary radiotherapy and chemotherapy-Radical and complete Excision	remission at 6 months
Yong-Soon Kwon [7]	2008	Clear cell carcinoma	Radical excision	no evidence of disease to 10 months
Current report	2014	Clear cell carcinoma	radical resection and chemotherapy	no evidence of disease to 6 months

## Conclusions

We report a case of clear cell carcinoma arising from episiotomy scar. There are only three clear cell carcinoma in episiotomy scar reported, no standard treatment established. Accumulation of management data on these rare tumors and Long-term follow-up of such patients is therefore important.

## Consent

Written informed consent was obtained from the patient for publication of this Case report and any accompanying images. A copy of the written consent is available for review by the Editor-in-Chief of this journal.
